# Editorial: Traumatic Brain Injury and Autoimmune Disease

**DOI:** 10.3389/fneur.2021.702431

**Published:** 2021-06-18

**Authors:** Sharon Baughman Shively, Braxton B. Wannamaker, Adam M. Willis, John F. Brugge, James Ness

**Affiliations:** ^1^Independent Researcher, Potomac, MD, United States; ^2^Department of Neurology, Medical University of South Carolina, Charleston, SC, United States; ^3^Department of Neurology, Brooke Army Medical Center, Fort Sam Houston, San Antonio, TX, United States; ^4^Department of Mechanical Engineering, Michigan State University, East Lansing, MI, United States; ^5^Department of Neuroscience, School of Medicine and Public Health, University of Wisconsin, Madison, WI, United States; ^6^Department of Behavioral Sciences and Leadership, United States Military Academy, West Point, NY, United States

**Keywords:** traumatic brain injury, autoimmune disease, chronic traumatic encephalopathy, posttraumatic stress disorder, blast wave, high explosive exposure, inflammation

The Guest Editors dedicate this article collection to Steven Edward Kornguth, PhD, who died September 21, 2020 after a brief illness. Steve was our colleague, collaborator, mentor, and friend. Early in his career, he conducted research in Tay-Sachs disease, Wilson's disease, multiple sclerosis, and myasthenia gravis, which eventually led to his reasoning that autoimmune mechanisms contribute to several neuropsychiatric ailments. Steve “retired” from the University of Wisconsin after 35 years and moved to Austin, Texas to be with family. In characteristic style, Steve immediately started to teach courses and lead a multi-university team in collaboration with the US Army Research Laboratory to conduct research related to autoimmunity, traumatic brain injury (TBI), kinesiology, and chronic traumatic encephalopathy (CTE). Among his projects, he organized this Research Topic (Figure 1).

**Figure 1:**
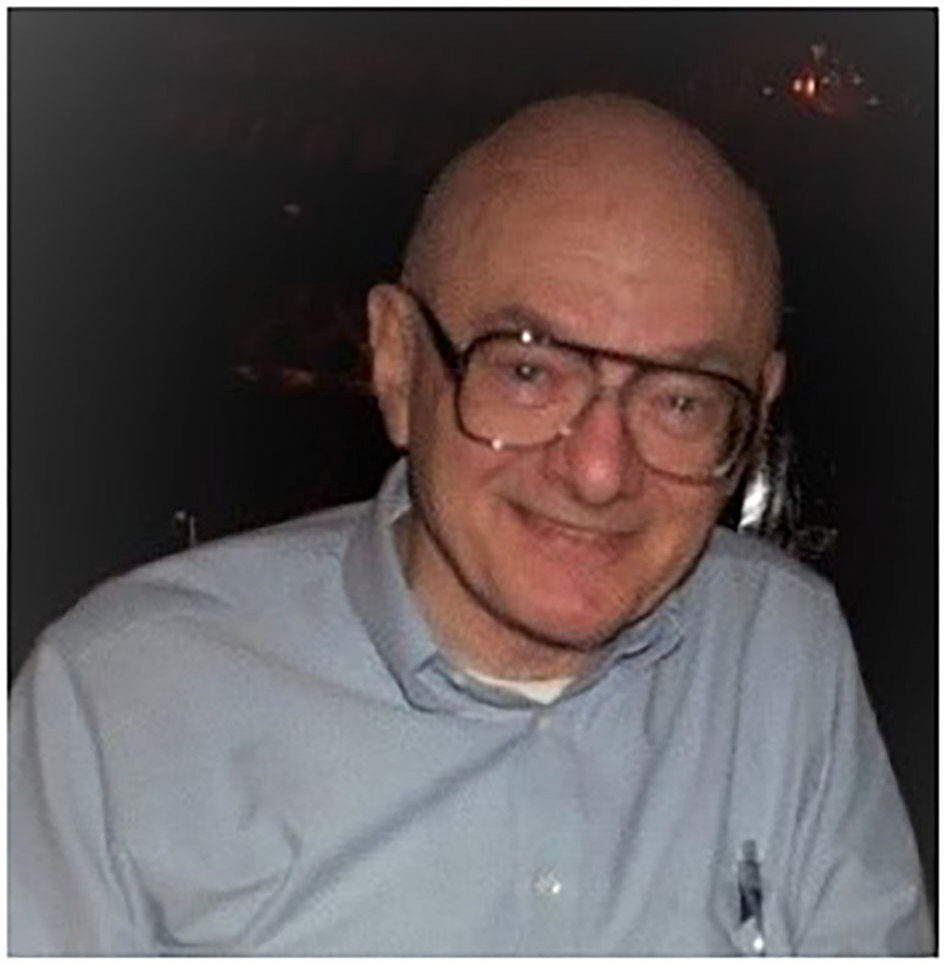
Steven Edward Kornguth, PhD (December 1, 1935–September 21, 2020) at a professional conference in 2009. Image credit: James Ness. Written informed consent was obtained for the publication of the image.

From 2000 to 2020, the Department of Defense (DoD) reported ~430,000 TBIs, with about 80% classified as mild (mTBI, concussion). Because US service members incur repetitive mTBIs (CTE risk factor) from contact sports and motor vehicle accidents, as well as military training and combat, the DoD and National Collegiate Athletic Association partnered to form the Concussion Assessment, Research and Education (CARE) Consortium, creating a national multisite research network to study the clinical and neurobiological natural history of mTBI (Houston et al.). The CARE Consortium provides a vast database invaluable to researching primary and secondary mechanisms after mTBI, with the goal of improving protection, diagnosis, treatment, return-to-activity/duty criteria, and neurodegenerative disease prevention. Particularly within the military population, mTBI and posttraumatic stress disorder (PTSD) frequently coexist.

With deployment overseas, military personnel also risk TBI from exposure to high explosive detonations, which generate blast waves. The mechanics and resultant physical brain injury patterns consequent to blast wave are poorly understood. Miller et al. conducted computational simulations using a human head model to test the hypothesis that blast wave causes damage at intracranial mechanical interfaces. Simulations revealed diffuse fluid cavitation within cerebrospinal fluid (CSF) juxtaposed to the brain parenchymal borders of subpial glial plate and periventricular tissues, which correspondingly showed local maxima of strain/strain rates. Simulations also revealed intravascular cavitation with similar apposed high perivascular tissue strain/strain rates within brain parenchyma. The association between high material strain/strain rates and fluid cavitation with brain injury requires experimentation for validation; interestingly, these simulated results generally corroborate interface astroglial scarring patterns in postmortem brain tissues from blast-exposed military personnel.

Development of reliable experimental systems producing blast waves representative of those from high explosives is essential to understanding potential consequences to the human brain. Kumar and Nedungadi analyzed compression-driven shock tubes, which generate blast waves with varying pressure waveforms, with computational fluid dynamics simulations. They studied effects of driver gas, driver (breech) length, and membrane burst pressure of a constant-area shock tube on pressure signatures. The authors also examined discrete locations in the shock tube to determine blast wave evolution in time at these points. The results provide guidelines for shock tube designs to produce desired blast profiles.

Steve contributed his own paper to this Topic. To test the hypothesis that CSF amplifies shear strain at neocortical sulcal depths during impact TBI (water hammer effect), Fagan et al. conducted simulations of frontal blunt impacts using two-dimensional finite element computational models of human heads with gyrencephalic brains. Unexpectedly, CSF was not driven into the sulci during impact; however, sulcal depths displayed maximum shear strains, which correlates with the injury patterns of microbleeds detected with magnetic resonance imaging (MRI) in contact sports athletes. The authors proposed that inflammatory and autoimmune processes lead to hyperphosphorylated *tau* in neurons, astrocytes, and neurites around small blood vessels at these injured sulcal depths (CTE lesion).

In their study, Edwards et al. addressed the association between neuroimaging and circulating proinflammatory cytokines within 24 h after mTBI (Glasgow Coma Scale >13). The researchers divided 250 mTBI participants into three cohorts: positive computed tomography (CT) findings, positive MRI but negative CT findings, and negative CT/MRI findings. Ultrasensitive immunoassay analyses of plasma showed interleukin 6, tumor necrosis factor alpha, and vascular endothelial growth factor elevation in patients of the CT+ and MRI+ groups in comparison to those of the CT–/MRI– group. The authors concluded that this serum biomarker panel may differentiate acute mTBI patients with positive vs. negative neuroimaging with further investigation.

Applying the connection between inflammation and neuropsychiatric clinical expression, Brenner et al. conducted a pilot trial to evaluate immunomodulatory probiotic therapy for US military veterans with concurrent mTBI and PTSD symptoms. Sixteen participants were treated with *Lactobacillus reuteri* DSM 17938 supplement daily for 8 weeks and 15 participants with placebo. The study showed feasibility, acceptability, and safety, and in the treatment group, decreases in plasma C-reactive protein concentrations and sympathetic pathway responses during social stress tests. These results support the rationale for a large trial focusing on inflammatory biomarkers and clinical outcomes for mTBI and PTSD patients.

For advancing treatment of severe TBI (sTBI, Glasgow Coma Scale 3–8) due to impact, Chen et al. performed a randomized, controlled clinical trial comparing intraoperative rapid decompression of intracranial pressure with craniectomy (conventional therapy, *n* = 124) vs. controlled decompression (*n* = 124) in adults aged 18–75 years old. The patients who underwent controlled decompression displayed improved clinical outcomes, as demonstrated with significantly reduced morbidity (intraoperative brain swelling, delayed hematoma) and mortality (30 days) in addition to significantly improved Extended Glasgow Outcome Scale scores (6 months) and relatively lower incidents of posttraumatic cerebral infarction. These results warrant pursuit of larger, multicenter, randomized controlled trials to determine effectiveness of controlled decompression surgery for sTBI patients.

This Research Topic highlights ongoing TBI research efforts through multiple approaches—from study of mechanics with physical loading on the human head and resultant brain injuries to discovery of biomarkers and conduction of clinical trials. A critical research objective is linkage of quantifiable doses from various head insults to specific brain injuries, resultant pathophysiological pathways, and associated neuropsychiatric sequelae. The particular hope Steve gave us is his research and leadership to understanding inflammatory and autoimmune mechanisms after TBI in relation to clinical expression and disease development.

## Author Contributions

SS, BW, AW, JB, and JN wrote sections of the manuscript. All authors contributed to manuscript revision and approved the submitted version.

## Conflict of Interest

The authors declare that the research was conducted in the absence of any commercial or financial relationships that could be construed as a potential conflict of interest.

